# Cell-Type Specific Changes in DNA Methylation of *SNCA* Intron 1 in Synucleinopathy Brains

**DOI:** 10.3389/fnins.2021.652226

**Published:** 2021-04-28

**Authors:** Jeffrey Gu, Julio Barrera, Young Yun, Susan K. Murphy, Thomas G. Beach, Randy L. Woltjer, Geidy E. Serrano, Boris Kantor, Ornit Chiba-Falek

**Affiliations:** ^1^Division of Translational Brain Sciences, Department of Neurology, Duke University Medical Center, Durham, NC, United States; ^2^Center for Genomic and Computational Biology, Duke University Medical Center, Durham, NC, United States; ^3^Division of Reproductive Sciences, Department of Obstetrics and Gynecology, Duke University Medical Center, Durham, NC, United States; ^4^Banner Sun Health Research Institute, Sun City, AZ, United States; ^5^Layton Aging and Alzheimer’s Disease Center, Department of Pathology, Oregon Health & Science University, Portland, OR, United States; ^6^Viral Vector Core, Duke University Medical Center, Durham, NC, United States; ^7^Department of Neurobiology, Duke University Medical Center, Durham, NC, United States

**Keywords:** α-synuclein gene (*SNCA*), dementia with Lewy body, Parkinson’s disease, DNA methylation, fluorescence-activated nuclei sorting, bisulfite pyrosequencing

## Abstract

Parkinson’s disease (PD) and dementia with Lewy body (DLB) are the most common synucleinopathies. *SNCA* gene is a major genetic risk factor for these diseases group, and dysregulation of its expression has been implicated in the genetic etiologies of several synucleinopathies. DNA methylation at CpG island (CGI) within *SNCA* intron 1 has been suggested as a regulatory mechanism of *SNCA* expression, and changes in methylation levels at this region were associated with PD and DLB. However, the role of DNA methylation in the regulation of *SNCA* expression in a cell-type specific manner and its contribution to the pathogenesis of PD and DLB remain poorly understood, and the data are conflicting. Here, we employed a bisulfite pyrosequencing technique to profile the DNA methylation across *SNCA* intron 1 CGI in PD and DLB compared to age- and sex-matched normal control subjects. We analyzed homogenates of bulk *post-mortem* frozen frontal cortex samples and a subset of neuronal and glia nuclei sorted by the fluorescence-activated nuclei sorting (FANS) method. Bulk brain tissues showed no significant difference in the overall DNA methylation across *SNCA* intron 1 CGI region between the neuropathological groups. Sorted neuronal nuclei from PD frontal cortex showed significant lower levels of DNA methylation at this region compared to normal controls, but no differences between DLB and control, while sorted glia nuclei exhibited trends of decreased overall DNA methylation in DLB only. In conclusion, our data suggested disease-dependent cell-type specific differential DNA methylation within *SNCA* intron 1 CGI. These changes may affect *SNCA* dysregulation that presumably mediates disease-specific risk. Our results can be translated into the development of the *SNCA* intron 1 CGI region as an attractive therapeutics target for gene therapy in patients who suffer from synucleinopathies due to *SNCA* dysregulation.

## Introduction

Synucleinopathies are a group of neurodegenerative disorders that share a pathological hallmark of intracellular inclusions, composed largely of the α-synuclein protein (SNCA), known as Lewy bodies (LBs) and Lewy-related neurites (Spillantini et al., 1997; Spillantini, 1999; Galvin et al., 2001; Jellinger, 2003; Marti et al., 2003). Genetic studies including GWAS for the most common synucleinopathies, Parkinson’s disease (PD) and dementia with LBs (DLB), have implicated *SNCA* gene as a highly significant genetic risk factor for these diseases (Pals et al., 2004; Mueller et al., 2005; Maraganore et al., 2006; Mizuta et al., 2006; Ross et al., 2007; Winkler et al., 2007; Myhre et al., 2008; Pankratz et al., 2009; Satake et al., 2009; Simon-Sanchez et al., 2009; Bras et al., 2014; Nalls et al., 2014). Although the precise mechanisms underlying the associations of *SNCA* with PD and DLB are yet to be discovered, accumulating evidence suggested that overexpression of *SNCA* may play a crucial role in etiology of these diseases (reviewed in Tagliafierro and Chiba-Falek, 2016). Thus, understanding the regulation of *SNCA* in health and disease will provide mechanistic insights into the genetic risk driven by the *SNCA* locus.

DNA methylation is an epigenetic mechanism of gene regulation, and several groups have studied the DNA methylation profiles of *SNCA* intron 1 region in relation to *SNCA* expression in the context of PD and DLB. Increased *SNCA* expression was found to be coincidental to demethylation of CpGs at *SNCA* intron 1 (Jowaed et al., 2010; Matsumoto et al., 2010; Wang et al., 2013). Analysis of *postmortem* brain tissues and blood from PD patients demonstrated lower methylation levels at *SNCA* intron 1 in several brain regions compared to control donors (Jowaed et al., 2010; Matsumoto et al., 2010; Desplats et al., 2011; Ai et al., 2014). DNA methylation changes at *SNCA* intron 1 and elevated mRNA levels were also reported in blood samples from DLB and AD patients (Funahashi et al., 2016; Yoshino et al., 2016). However, other reported no significant differences in the DNA methylation levels within *SNCA* intron 1 in *postmortem* brain tissues from PD vs controls (Guhathakurta et al., 2017). These conflicting results may stem from several reasons such as sample size, the analyzed tissue type and/or brain structure, and the sensitivity and accuracy of the method used for profiling the DNA methylation. Nonetheless, one limitation common to all previous studies is the use of bulk brain tissues (brain tissue homogenates) for the analysis. The brain tissue is heterogeneous and composed of multiple cell types which makes it difficult to assess molecular characteristics of individual cell types. Furthermore, the heterogeneity also introduces technical shortcoming related to sample-to-sample differences in cell-type composition of the brain tissue. The variability in the proportion of the different cell types is even more pronounced in samples from brains affected by neurodegeneration.

**FIGURE 1:**
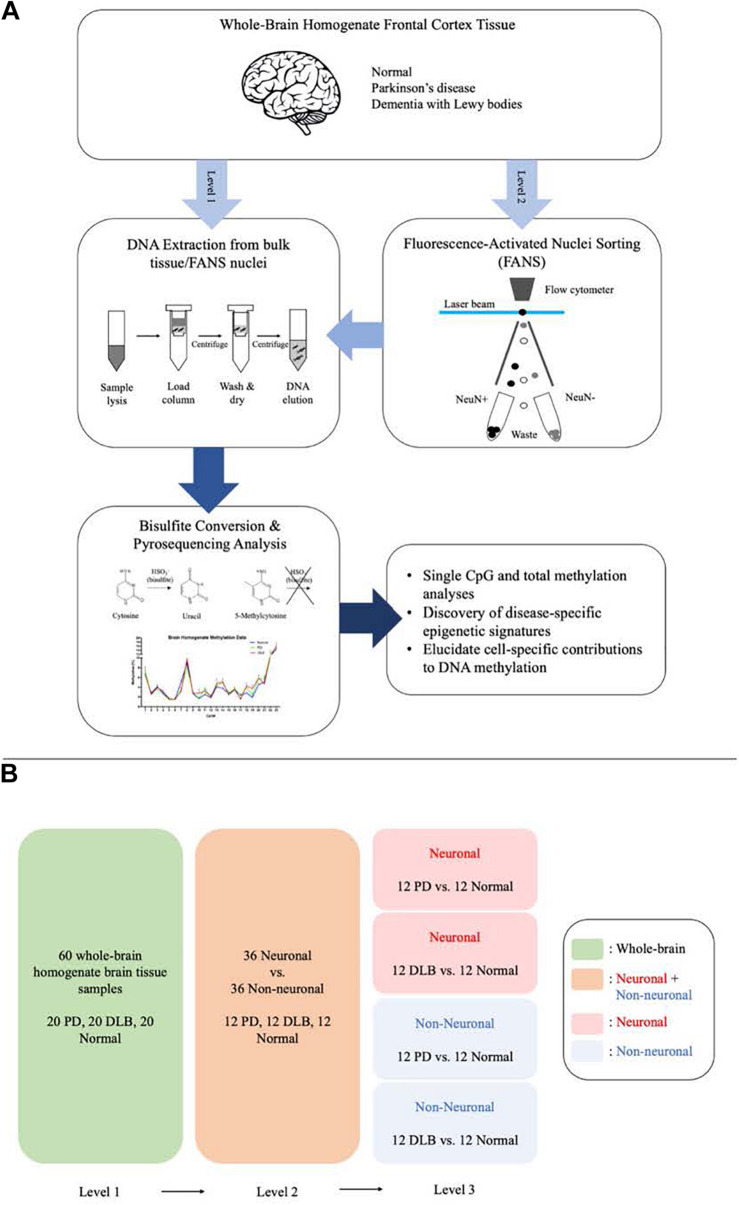
Schematic presentation of the study workflow and comparisons. **(A)** The study workflow. **(B)** The comparison levels.

We designed this study to further explore the DNA methylation profiles across *SNCA* intron 1 in human frontal cortex and to characterize PD- and DLB-associated changes in the DNA methylation levels in neurons and glia cells. To this end, we utilized the bisulfite pyrosequencing method for quantitative measurement of DNA methylation levels at individual CpG sites in *SNCA* intron 1. We employed the pyrosequencing method using bulk brain tissues and fluorescence-activated nuclei sorting (FANS)-sorted neuronal and non-neuronal nuclei (Figure 1A), and compared in three levels the overall DNA methylations levels across *SNCA* intron 1 CpG island (CGI) and the methylation profiles at individual CpG sites (Figure 1B). The outcomes of this study pointed at brain cell type-specific differences in the overall DNA methylation levels and in the methylation at individual CpG sites across *SNCA* intron 1 CGI that are specific to either PD or DLB.

## Materials and Methods

### Study Samples

The study cohort for the bulk brain tissues analysis (*N* = 60) consisted of individuals with three autopsy-confirmed neuropathological diagnoses: (1) PD (*N* = 20); (2) DLB (*N* = 20); and (3) clinically and neuropathologically normal subjects (*N* = 20) (Table 1). Frontal cortex brain tissues were obtained through the *Kathleen Price* Bryan *Brain Bank (KPBBB)* at Duke University, the Banner Sun Health Research Institute Brain and Body Donation Program, and Layton Aging & Alzheimer’s Disease Center at Oregon Health and Science University. Neuropathologic phenotypes were determined in *postmortem* examination following standard well-established methods following the method and clinical practice recommendations of McKeith and colleagues (McKeith et al., 1999, 2005). The density of the LB pathology (in a standard set of brain regions) received scores of mild, moderate, severe, and very severe. The neurologically healthy brain samples were obtained from *postmortem* tissues of clinically normal subjects who were examined, in most instances, within 1 year of death and were found to have no cognitive disorder or parkinsonism and neuropathological findings insufficient for diagnosing PD, Alzheimer’s disease (AD), or other neurodegenerative disorders. All samples were whites. *Demographics for these subjects are summarized in Table 1. The analysis was repeated using sorted nuclei (by NeuN-FANS, see below the next section in the methods) isolated from a subset of this cohort (N = 36), 12 samples from each pathological category (PD, DLB, and normal control).* The project was approved by the Duke Institution Review Board (IRB protocol numbers Pro00053335, Pro00028081, and Pro00010141). The methods were carried out *in accordance with* the relevant guidelines and regulations.

**TABLE 1:** Demographic description of the study cohorts.

	**PD**	**DLB**	**Normal control**
**Bulk tissue cohort**			
*Total number*	20	20	20
*Caucasian %*	100	100	100
*Male %*	65	55	55
*Age at death (mean ± SEM)*	76.0 ± 1.4	76.9 ± 1.6	80.6 ± 1.4
*Post-mortem interval (mean ± SEM)*	5.65 ± 1.4	8.95 ± 2.4	5.89 ± 1.5
**FANS-sorted nuclei sub-cohort**			
*Total number*	12	12	12
*Caucasian %*	100	100	100
*Male %*	50	50	50
*Age at death (mean ± SEM)*	78.3 ± 1.7	77.7 ± 1.5	82.8 ± 1.7
*Post-mortem interval (mean ± SEM)*	6.26 ± 2.1	6.19 ± 3.0	7.58 ± 2.3

*Abbreviations: DLB, dementia with Lewy bodies; PD, Parkinson’s disease; SEM, standard error of the mean.*

### Fluorescence-Activated Nuclei Sorting

#### Tissue Dissociation and Nuclei Extraction

Methods were performed according to established protocols (Marzluff, 1990; Jiang et al., 2008) with some modifications. Briefly, 50 mg of frozen frontal cortex was thawed for 10 min on ice in lysis buffer (0.32 M sucrose, 5 mM CaCl2, 3 mM magnesium acetate, 0.1 mM EDTA, 10 mM Tris-HCl pH8, 1 mM DTT, and 0.1% Triton X-100). The tissue was gently dissociated and homogenized in a 7 ml dounce tissue homogenizer (Corning) with approximately 25 strokes of pestle A in 45 s, and then filtered through a 100-μm cell strainer. The filtered lysate was transferred to a 14 × 89 mm polypropylene ultracentrifuge tube, carefully underlaid with sucrose solution (1.8 M sucrose, 3 mM magnesium acetate, 1 mM DTT, and 10 mM Tris-HCl, pH 8), and subjected to ultracentrifugation at 107,000 RCF for approximately 30 min at 4°C. Supernatant and the debris interphase were carefully aspirated, and 100 μl PBS (-Mg2+ and -Ca2+) was added to the nuclei pellet. After a 5-min incubation on ice, nuclei were gently resuspended and transferred to a 1.5-ml polypropylene microcentrifuge tube for staining.

#### Immunostaining of Nuclei

Nuclei were stained in 0.05% BSA, 1% normal goat serum, DAPI (1 μg/ml), and PE-conjugated anti-NeuN antibody (1:125, Millipore FCMAB317PE) in PBS (-Mg2+ and -Ca2+), in the dark for 30 min at 4°C. A DAPI-only control was prepared to set gates for sorting. After staining, nuclei were filtered through a 40-μm cell strainer into a polypropylene round-bottom 5-ml tube and sorted.

#### Immunofluorescence Microscopy

After homogenization and sucrose gradient ultracentrifugation, a portion of the nuclei was counted, resuspended in 4% PFA, stained, plated on 12 mm coverslips at 10,000 nuclei per coverslip, incubated 20 min at room temperature, mounted, and imaged on a confocal microscope.

#### Fluorescence-Activated Nuclei Sorting (FANS) of Neuronal and Non-neuronal Nuclei

Sorting was performed using a MoFlo Astrios flow cytometer (Beckman Coulter) equipped with a 70-μm nozzle, operating at 35 psi. Standard gating procedures were used. Briefly, the first gate allowed separation of intact nuclei from debris. The second gate allowed us to identify individual nuclei, and exclude doublets and other aggregates. The third and fourth gates distinguish between PE+ and PE− nuclei and allowed us to sort and separate NeuN+ nuclei from NeuN− nuclei. Nuclei were sorted into 1 ml PBS (−Mg2+ and −Ca2+) in a 2-ml polypropylene tube pre-coated with 200 μl of 5% BSA and rotated at 20 rpm at 4°C.

### DNA Extraction From Bulk Tissue and FANS Nuclei

Genomic DNA (gDNA) extraction was performed using DNeasy Blood and Tissue Kit (QIAGEN) per the manufacturer’s instructions. Nucleic acid quality and concentration were evaluated using a NanoDrop 8000 spectrophotometer (ThermoFisher).

### Bisulfite Pyrosequencing

Bisulfite pyrosequencing was performed as previously described (Kantor et al., 2018). Briefly, 800 ng of gDNA was treated with sodium bisulfite using the Zymo EZ DNA Methylation Kit (Zymo Research). Pyrosequencing assays were designed using PyroMark assay design software version 1.0.6 (Biotage; Uppsala, Sweden) to specifically evaluate the methylation levels of the 23 CpG sites in the *SNCA* intron 1 region (chr4: 89,836,150–89,836,593 [GRCh38/hg38]) (Supplementary Figure 1A). These assays were validated for linearity and range on a PyroMark Q96 MD pyrosequencer using mixtures of various ratios of unmethylated and methylated bisulfite-modified DNA (Supplementary Figure 1B). Bisulfite modified DNA (20 ng) was added to the PyroMark PCR Master Mix (Qiagen) and subjected to PCR using the following conditions: 95°C for 15 m, 50 cycles of 94°C for 30 s, 56°C for 30 s, and 72°C for 30 s with a final 10 m extension step at 72°C. Supplementary Table 1 lists the forward, reverse, and sequencing primers of the seven assays that were designed, and the specific CpG sites that each assay covers. Pyrosequencing was conducted using PyroMark Gold Q96 Reagents (Qiagen) following the manufacture’s protocol. Methylation values for each CpG site were calculated using Pyro Q-CpG software 1.0.9 (Biotage). Each gDNA sample was analyzed in two independent experiments.

### Statistical Analysis

Individual CpG methylation averages were calculated for all samples in each pathology group. Outlier analysis was performed for each CpG site across all samples. The range for outlier values was calculated using Tukey’s fences:

$$\left[Q_{1}-k\,\left(Q_{3}-Q_{1}\right),Q_{3}+k\,\left(Q_{3}-Q_{1}\right)\right]$$

where *Q*_1_ and *Q*_3_ are the first and third quartiles, and *k* is a non-negative constant. We used *k* = 1.5 to indicate outliers. Any values falling outside this range (11% of the bulk tissues data points and 9% of the sorted nuclei data points) were excluded from the average and subsequent analyses.

One-way ANOVA followed by Dunnett’s *post hoc* test for multiple comparisons were performed between the three pathology groups (PD and DLB vs Normal) for individual CpG sites and total methylation averages. Unpaired *t*-tests were performed between neuronal and glia for individual CpG sites and total methylation averages. All analyses were performed using Microsoft Excel and GraphPad Prism.

## Results

### DNA Methylation Levels at the *SNCA* Intron 1 CGI in Bulk Brain Tissue Homogenates From PD and DLB Compared to Control

We profiled the DNA methylation at the CGI located within intron 1 of *SNCA* gene using bulk frontal cortex samples from 20 PD, 20 DLB, and 20 normal control (Table 1 and level 1, Figure 1). The DNA methylation profiles demonstrated that this region is overall hypomethylated.

**FIGURE 2:**
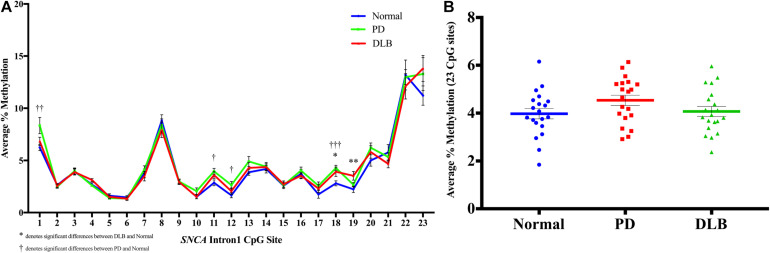
DNA methylation profiles of the *SNCA* intron 1 CGI in the bulk frontal cortex tissues. DNA from each bulk frontal cortex sample was bisulfite converted, and the methylation (%) of the individual CpGs was quantitatively determined by pyrosequencing. **(A)** Graph depicts the average percentage of DNA methylation level for 20 PD, 20 DLB, and 20 matched normal control subjects at each of the 23 CpG sites within the *SNCA* intron 1 CGI. **(B)** Box-and-scatterplot chart displays for each subject the DNA methylation average percentages across all 23 CpG sites within the *SNCA* intron 1 CGI [Chr4: 89,836,150-89,836,593 (GRCh38/hg38)] (denoted by colored points). The box plot also depicts the mean (denoted by colored horizontal line) and upper and lower quartiles (denoted by gray thin lines) of DNA methylation average percentages across all 23 CpG sites for all subjects in each group of PD, DLB, and normal control. The significance of the differences in % of methylation was determined using one-way ANOVA followed by Dunnett’s *post hoc* test and denoted by asterisks (^∗^) for DLB vs normal, and by obelisks (†) for PD vs normal comparisons, with ^∗^/†*p* 0.05, ^∗∗^/††*p* 0.01, and ^∗∗∗^/†††*p* 0.005.

First, we analyzed the DNA methylation levels at individual CpG and found statistically significant differences at several CpG sites between the disease groups and normal control. Specifically, CpG site at position 18 showed significant higher levels of DNA methylation in both PD and DLB samples compared to the control group (*p* = 0.005 and 0.03, respectively), while at CpGs 1, 11, and 12, the DNA methylation levels were significantly elevated in PD only compared to control (*p* = 0.01, 0.02, and 0.04, respectively), and at CpG 19 a significant increase was observed in DLB only (*p* = 0.01) (Figure 2A). Next, comparisons of the overall DNA methylation levels at the CGI of *SNCA* intron 1, *i.e.*, the average across all 23 CpGs examined within the CGI, showed no significant difference between the neuropathological groups. However, a trend towards hypermethylation was detected in the PD samples compared to control samples (<20% increase; *p* = 0.1, Figure 2B). Noteworthy, the general trends of hypermethylation in PD and DLB were in contrary to previous reports (Jowaed et al., 2010; Matsumoto et al., 2010; Desplats et al., 2011; Ai et al., 2014; Funahashi et al., 2016; Yoshino et al., 2016) and unexpected since hypermethylation at promoter/intron 1 CGIs is generally associated with decreased gene expression, whereas overexpression of *SNCA* has been implicated in disease etiology.

### Neuronal vs Glia Cells in Frontal Cortex From PD, DLB, and Control

**FIGURE 3:**
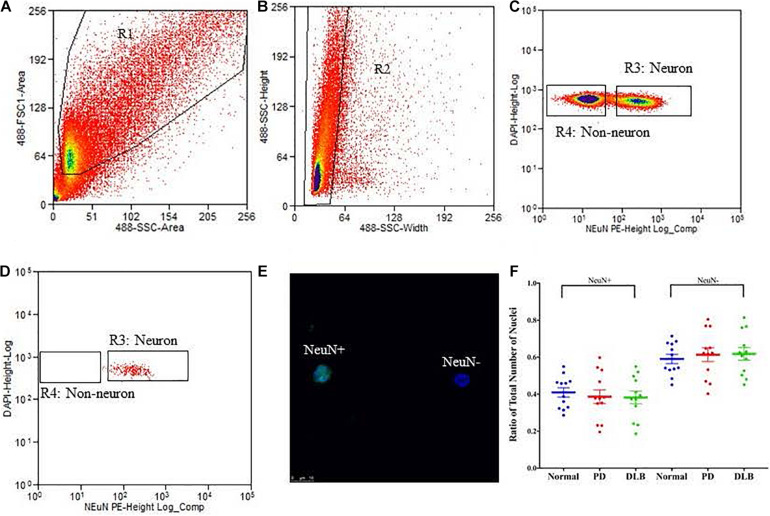
Isolation of nuclei from frozen brain samples. Human postmortem frontal cortex was dissociated; nuclei were isolated and stained with the nuclear stain DAPI and a monoclonal NeuN antibody conjugated to PE. The panels represent an example of a single sort experiment performed using a particular brain donor (subject ID 1690). **(A)** Nuclei were first sorted based on their forward and side scatter from all possible events (R1 gate). **(B)** Single nuclei were further sorted based on their size from the doublets or larger clumps of nuclei (R2 gate). **(C)** DAPI positive single cells were gated as either NeuN-PE positive (neurons, R3 gate) or NeuN-PE negative (glia, R4 gate). **(D)** Post-sort data showing the purity (>97%) of the separation between neuronal and non-neuronal nuclei. **(E)** Fluorescence image showing unsorted nuclei stained for NeuN (green) and DAPI (blue). The scale bar represents 10 μm. **(F)** Box-and-scatterplot chart of the ratios of neuronal (NeuN+) and non-neuronal nuclei (NeuN-) from total nuclei for each neuropathological group. Each point represents the ratio for an individual sample. The box denoted the mean (thick horizontal line) and upper and lower quartiles (thin shorter lines).

We applied the FANS method to separate neuronal and non-neuronal nuclei from a subset (12 PD, 12 DLB, and 12 control) of the archived frozen frontal cortex samples (Tables 1, 2). The non-neuronal cells population, *i.e.*, glia, are composed primarily of astrocytes, oligodendrocytes, and microglia, and to a lesser extent, nucleated blood cells and vascular cells. Nuclei were isolated from the frozen brain tissues, labeled with the monoclonal anti-NeuN antibody that recognizes specifically the neuronal nuclei, and subsequently the labeled vs unlabeled nuclei were sorted (Figures 3A–C). We confirmed the purity of the sorted neuronal and glial nuclei populations by repeating the flow on a small amount of the sorted nuclei and measuring the percentage of NeuN positivity and negative nuclei. This post-sort analysis demonstrated a sufficient level of separation with a purity of > 97% in the NeuN+ and NeuN- nuclei populations (Figure 3D). The specificity of the nuclei staining with the nuclear membrane marker, NeuN, was confirmed by immunofluorescence (Figure 3E).

**TABLE 2:** FANS neuronal and glia nuclei.

	**PD**	**DLB**	**Normal control**
*Total number*	12	12	12
*NeuN+ (%* ± *SEM)*	38.7 ± 3.74	38.2 ± 3.40	40.9 ± 2.52

Neurodegenerative processes in PD and DLB involved neuronal loss and gliosis. Next, we analyzed the flow cytometry data to determine the percentage of neuronal and glial nuclei from total nuclei for each frontal cortex sample. The ratios were variable across the individual samples (Figure 3F). However, there were no significant differences in the average percentages of neuronal and glia nuclei in the PD and DLB groups compared to the normal control group (*p* = 0.6 and 0.5, respectively, Figure 3F and Table 2).

### DNA Methylation Levels at the *SNCA* Intron 1 CGI in Neuronal Compared to Glia Sorted Nuclei

**FIGURE 4:**
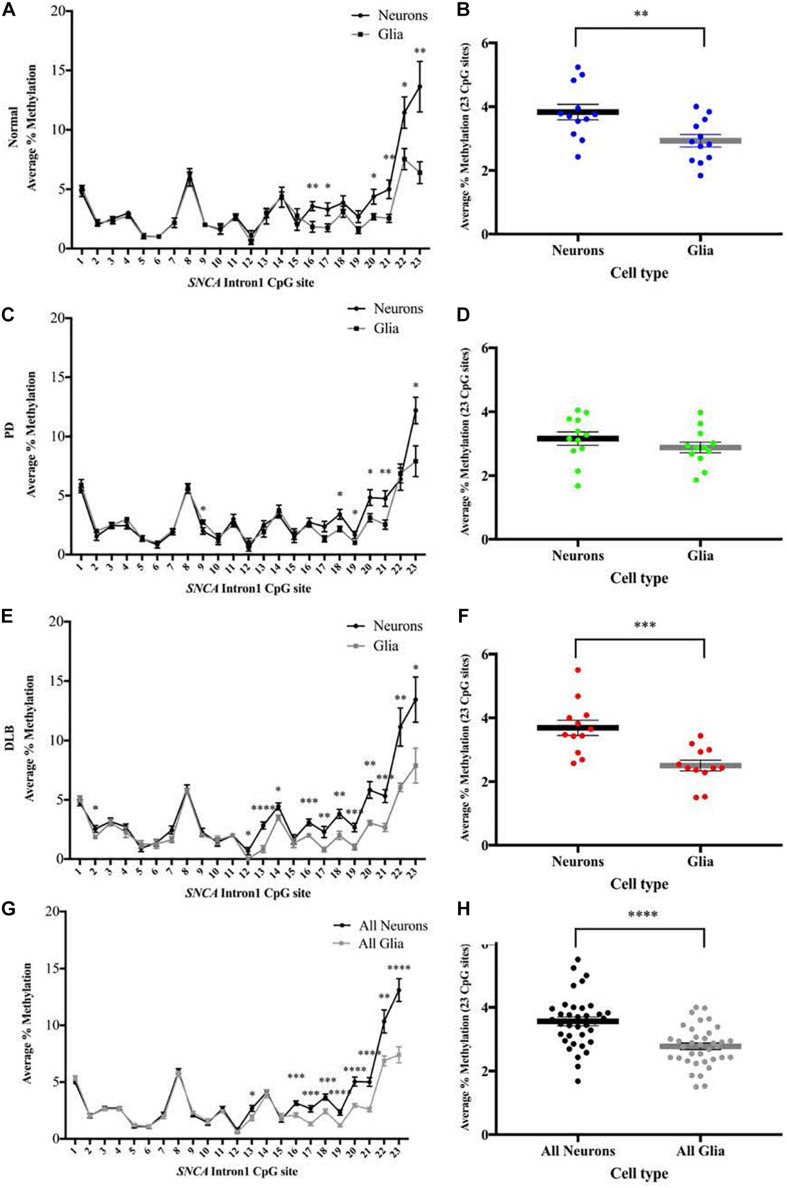
DNA methylation profiles of the *SNCA* intron 1 CGI in neuron vs glia from frontal cortex tissues. DNA from each sorted nuclei sample was bisulfite converted, and the methylation (%) of the individual CpGs was quantitatively determined by pyrosequencing. Left: Graphs depict the average percentage of DNA methylation level at each of the 23 CpG sites within *SNCA* intron 1 CGI from **(A)** 12 normal, **(C)** 12 PD, **(E)** 12 DLB, and **(G)** all 36 subjects. Box-and-scatterplot charts display for each sorted neuronal and glia nuclei sample from each subject the DNA methylation average percentages across all 23 CpG sites within the *SNCA* intron 1 CGI [Chr4: 89,836,150-89,836,593 (GRCh38/hg38)] (denoted by point). The box plots also depict the mean (denoted by thick horizontal lines) and upper and lower quartiles (denoted by thin lines) of DNA methylation average percentages across all 23 CpG sites for all sorted neuronal and glia nuclei samples from **(B)** 12 normal, **(D)** 12 PD, **(F)** 12 DLB, and **(H)** all 36 subjects. The significance of the differences in % of methylation was tested using the Student’s *t*-test and denoted by asterisks (^∗^) for neuron vs glia comparisons, with ^∗^*p* < 0.05, ^∗∗^*p* < 0.01, ^∗∗∗^*p* < 0.001, and ^****^p < 0.0001.

We used the sorted neuronal (NeuN+) and glial (NeuN-) nuclei to investigate the cell type-specific DNA methylation profiles at the CGI within *SNCA* intron 1. Following bisulfate conversion, DNA extracted from nuclei samples of the subset sample of 12 PD, 12 DLB, and 12 matched normal control (Table 1) were subjected to PCR amplification and pyrosequencing to quantify the methylation levels. Level 2 comparison (Figure 1B) showed that generally the methylation levels were higher in neuronal compared to those of glia cells and were significantly increased in the entire cohort and in the DLB and normal groups (Figure 4). Of note, in each of the three neuropathological groups, the increased levels of DNA methylation in neurons vs glia nuclei were greater and reached significance at the 3′ end of the CGI, specifically at CpG positions 16–23 (Figure 4). Collectively, our results suggested that the overall methylation levels in neurons are higher than in glia in both disease and control samples, consistent with a previous study which demonstrated that the levels of DNA methylation in the neurons are higher compared to glia (Coppieters et al., 2014).

### Neuron and Glia-Specific DNA Methylation Levels at the *SNCA* Intron 1 CGI in PD and DLB Compared to Control

**FIGURE 5:**
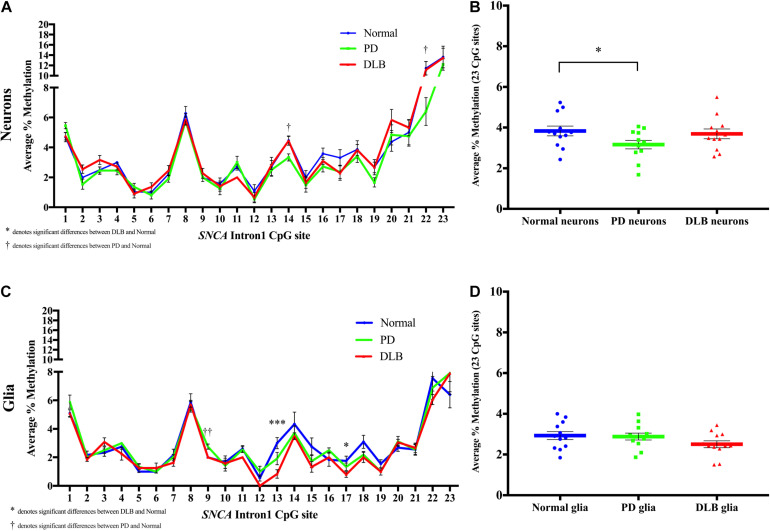
DNA methylation profiles of the *SNCA* intron 1 CGI in the sorted neuronal and glia nuclei from frontal cortex tissues. DNA from each neuronal and glia sorted nuclei sample was bisulfite converted, and the methylation (%) of the individual CpGs was quantitatively determined by pyrosequencing. Graph depicts the average percentage of DNA methylation level at each of the 23 CpG sites within the *SNCA* intron 1 CGI for **(A)** neuronal nuclei and **(C)** glia nuclei from 20 PD, 20 DLB, and 20 matched normal control subjects. The significance of the differences in % of methylation was determined using one-way ANOVA followed by Dunnett’s *post hoc* test and denoted by asterisks (^∗^) for DLB vs normal, and by obelisks (†) for PD vs normal comparisons, with ^∗^/†*p* < 0.05, ^∗∗^/††*p* < 0.005, and ^∗∗∗^/†††*p* < 0.001. Box-and-scatterplot charts display the DNA methylation average percentages across all 23 CpG sites within the *SNCA* intron 1 CGI [Chr4: 89,836,150-89,836,593 (GRCh38/hg38)] for each **(B)** neuronal and **(D)** glia nuclei isolated and sorted from each subject (denoted by colored points). The box plots also depict the mean (denoted by colored horizontal lines) and upper and lower quartiles (denoted by gray thin lines) of DNA methylation average percentages across all 23 CpG sites for all **(B)** neuronal and **(D)** glia sorted nuclei in each group of PD, DLB, and normal control. The significance of the differences in % of methylation was determined using Student’s *t*-test and repeated using one-way ANOVA followed by Dunnett’s *post hoc* test. ^∗^indicates *p* < 0.05 for *t*-test; upon applying the Dunnett’s multiple comparisons method, the result did not reach statistical significance (*p* = 0.08).

Next, we compared the DNA methylation profiles at *SNCA* intron 1 CGI between PD, DLB, and normal control within each cell-type, neuronal and glia (level 3, Figure 1B). Analysis of the DNA methylation levels at individual CpGs showed cell-type specific statistically significant changes at several CpG sites associated with either PD or DLB. For example, in neuronal nuclei from PD, CpG sites at the 3’ end of the CGI showed trends of hypomethylation compared to normal control, specifically, positions 14 and 22 exhibited significant lower levels of DNA-methylation in PD neurons (*p* = 0.02, Figure 5A). These trends were not observed in DLB neurons (Figure 5A). On the other hand, analysis of the glia nuclei did not detect significant hypomethylation at individual CpG sites in PD. However, in DLB glia, CpGs 13 and 17 showed significant decrease in methylation in comparison to control (*p* = 0.0008 and 0.04, respectively, Figure 5C).

We then calculated the average across all 23 CpGs comprising the CGI to determine the overall DNA methylation levels in this region. While the analysis of the neuronal nuclei showed no significant differences in the overall DNA methylation level between the DLB and the control groups, borderline significant lower overall DNA methylation levels were observed in neuronal nuclei from PD subjects compared to control (*p*_*t*__–test_ < 0.05, *p*_Dunnett_ = 0.08, Figure 5B). The glial cell population showed no significant differences in the overall DNA methylation levels in this region between all three neuropathological groups, although DLB glia exhibited a suggestive trend of overall decreased methylation (Figure 5D).

## Discussion

In this study, we found for the first-time disease-dependent cell type-specific differential DNA methylation profiles in the CGI located in *SNCA* intron 1. We demonstrated that PD and DLB did not share the same cell type-specific DNA methylation signatures, both at the overall DNA methylation level across the CGI and at individual CpG sites. Interestingly, this CGI was significantly hypomethylated in neurons sorted from PD brains and showed similar trends of lower DNA methylation levels in glia from DLB compared to controls. These results suggested that alterations in DNA methylation within *SNCA* intron 1 CGI may play distinctive functional roles in PD and DLB pathologies.

The role of demethylation of CpGs within *SNCA* intron 1 in the etiology of PD and DLB mediated by *SNCA* dysregulation was previously studied (Jowaed et al., 2010; Matsumoto et al., 2010; Desplats et al., 2011; Ai et al., 2014; Funahashi et al., 2016; Yoshino et al., 2016). Most of these studies used brain tissue homogenates that consist of multiple cell types; thus, it has been difficult to interpret the specific brain cell type responsible for the disease associated change in the DNA methylation pattern of *SNCA* intron 1 CGI. While PD and DLB primarily affect neurons, both neuronal and glia cells play important though distinctive roles in neurodegeneration and in the pathophysiology of PD and DLB (Tremblay et al., 2019; Kuter et al., 2020). Furthermore, epigenomes are known to differ between brain cell types (Iwamoto et al., 2011; Davies et al., 2012; Kozlenkov et al., 2014), with neurons holding higher global levels of DNA methylation than glial cells (Coppieters et al., 2014). As such, it is possible that DNA methylation differences between disease and normal states are cell type-specific in nature. The regulation of *SNCA* in homogenous populations of each particular cell type relevant to disease processes has been understudied. In this study, sorting neuronal from glia nuclei allowed us to detect distinct cell type-specific changes in DNA methylation profiles in PD vs DLB.

Previous studies investigating the DNA methylation profiles at *SNCA* intron 1 CGI generated ambiguous conclusions (Guhathakurta et al., 2017). Utilizing different tissues (brain vs blood and distinct brain regions) and technical shortcomings may explain, at least in part, some of the inconsistency. As discussed above, these studies analyzed bulk brain tissues which introduce biases related sample-to-sample variations in the proportion of each brain cell type. For example, while Jowaed et al. found overall hypomethylation of *SNCA*-intron 1 in all three brain regions, sub nigra, putamen, and cortex, of PD patients compared to control, we did not detect significant difference in the cortex homogenates from PD patients. Analysis of neuronal vs glia sorted nuclei allowed us to circumvent the limitation. Also, these studies utilized older cloning-to-sequencing protocols followed by bisulfite conversion, which are known to be prone to undesirable biases associated with the clonal enrichment during bacterial expansion phase. To overcome this issue, we used pyrosequencing. Overall, the outcomes of our study addressed the inconsistency regarding the role of DNA methylation within *SNCA*-intron 1 in synucleinopathies.

Our study has some limitations. First, of our study sample cohorts, especially the sorted nuclei, were relatively small. Thus, the cell type-specific disease-dependent associations warrant further investigations in a larger sample size to confirm the observed trends. Second, the analyses identified disease associated DNA methylation profiles in specific brain cell type; however, whether these epigenetic alterations are causative or a consequence of neurodegenerative process in PD or DLB is currently unknown and the underpinning mechanistic understanding is yet to be explored. Third, the exact neuronal and glia sub-type driving the changes in DNA methylation profiles require finer resolution using single-cell omics technologies. Last, while studying the correlation between changes in DNA methylation profiles and *SNCA* differential expression is interesting, the current study focused on disease associated changes in DNA-methylation. Future studies using a larger sample size will apply advanced single-cell genomic technologies to investigate the cell-type specific multi-omics relationships across the *SNCA* genomic region, including DNA methylation levels, chromatin state, and gene expression in the context of synucleinopathies.

This study further supports the development of *SNCA* intron 1 CGI as an attractive therapeutics target for synucleinopathies. Indeed, we recently demonstrated that DNA hypermethylation targeted at *SNCA* intron 1 CGI resulted in downregulation of *SNCA* mRNA and protein and rescued disease-related cellular phenotypes human-induced pluripotent stem cell (hiPSC)-derived dopaminergic neurons from a PD patient with *SNCA* triplication. The induction of DNA methylation targeted at the hypomethylated *SNCA* CGI was achieved by an innovative gene therapy approach based on a lentivirus-delivered dCas9-*de novo* methyltransferase 3A (DMNT3A) and demonstrated promising results for further validations in preclinical and clinical settings.

## Data Availability Statement

The original contributions presented in the study are included in the article/Supplementary Material, further inquiries can be directed to the corresponding author/s.

## Ethics Statement

The studies involving human participants were reviewed and approved by the Duke Institution Review Board (IRB). The methods were carried out in accordance with the relevant guidelines and regulations. Written informed consent for participation was not required for this study in accordance with the national legislation and the institutional requirements.

## Author Contributions

JG: methodology, investigation, formal analysis, visualization, and writing—original draft. JB: methodology, investigation, visualization, and writing—original draft. YY: methodology and investigation. SM: reagents, data generation, and writing—review and editing. TB, RW, and GS: reagents. BK: conceptualization, writing—original draft, and writing—review and editing. OC-F: conceptualization, supervision, obtained funding, writing—original draft, and writing—review and editing. All authors contributed to the article and approved the submitted version.

## Conflict of Interest

OC-F and BK are inventors of intellectual property PCT/US2019/028786 which is related to this research and is licensed to Seelos Therapeutics, Inc., Duke, the inventors, and Seelos Therapeutics, Inc., could potentially benefit from the outcome of this research if commercially successful. The remaining authors declare that the research was conducted in the absence of any commercial or financial relationships that could be construed as a potential conflict of interest.
