# Supplementary material for: Cell-Type Specific Changes in DNA Methylation of SNCA Intron 1 in Synucleinopathy Brains
Source: Front Neurosci. 2021 Apr 28;15:652226. doi: 10.3389/fnins.2021.652226 (PMC8113398; doi:10.3389/fnins.2021.652226)
Supplement: Supplementary file 1 [file Table_1.DOCX]

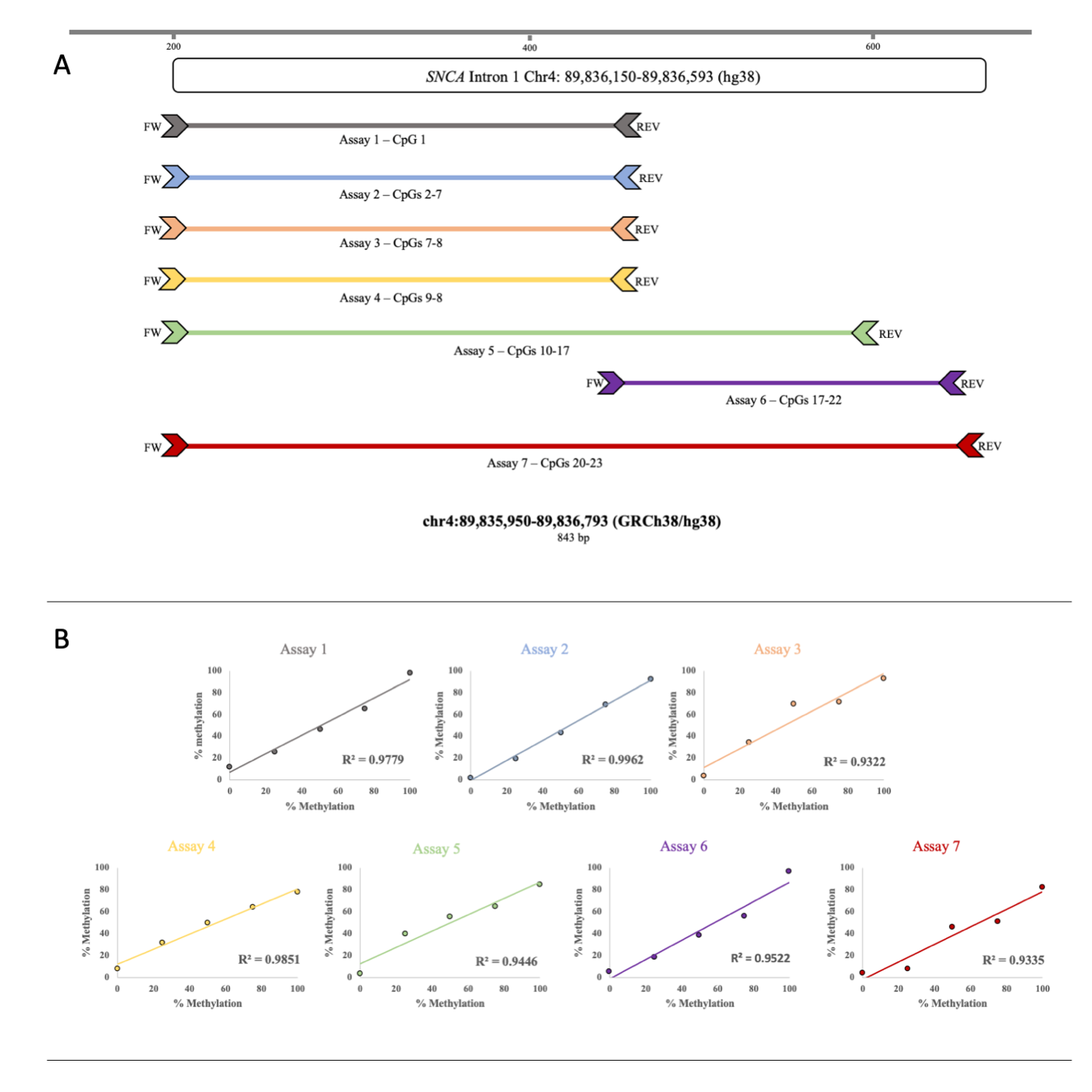


**Supplementary Figure 1. Pyrosequencing assays design and validation**

(**A**) The seven assays designed to cover all 23 CpGs across the 444 bp CGI region within *SNCA* intron 1. (**B**) The results of the validation tests of the seven assays utilized in the DNA-methylation profile analyses reported in this study. The designed assays were validated for linearity and range using the following ratios of unmethylated (U) and methylated (M) bisulfite-converted DNA: 100U:0M, 75U:25M, 50U:50M, 25U:75M, 0U:100M (EpiTect Control DNA Set; Qiagen).

**Supplementary Table 1. Pyrosequencing assay primers and CpG coverage**
